# Supplementary material for: Nasopharyngeal carriage of Streptococcus pneumoniae among Brazilian children: Interplay with viral co-infection
Source: PLoS One. 2025 Jan 2;20(1):e0316444. doi: 10.1371/journal.pone.0316444 (PMC11694996; doi:10.1371/journal.pone.0316444)
Supplement: S1 Table — (PDF) [file pone.0316444.s001.pdf]

S1 Table - Primers and probes used in qPCR assays

Target	Sequence (5´-3´)	nM	Target gene	GenBank no.	References
18S	18S-F GGAGTATGGTTGCAAAGCTGA	300	18S ribosomal RNA	MK533682.1	[1]
	18S-R GGTGAGGTTTCCCGTGTTG	300			
	Cy5-AAGGAATTGACGGAAGGGCA-BHQ3	250			
FLU A	FLUA-F: CAAGACCAATCYTGTCACCTCTGAC	600	Matrix protein gene	OK631772.1	[2]
	FLUA-F: CAAGACCAATYCTGTCACCTYTGAC	600			
	FLUA-R: GCATTYTGGACAAAVCGTCTACG	600			
	FLUA-R: GCATTTTGGATAAAGCGTCTACG	600			
	FLUA-FAM: TGCAGTCCT/ZEN/CGCTCACTGGGCACG	250			
FLU B	FLUB-F: GTCCATCAAGCTCCAAGTTTT	400	NEP gene	OK484400.1	[2]
	FLUB-R: TCTTCTTACAAGCTTGCTTGC	600			
	FLUB-VIC: CCAATTCGA/ZEN/GCAGCTGAAACTGCGGTG	200			
ADV	ADV-F: GCCACGGTGGGGTTTCTAAACTT	400	Exon gene	MK994980.1	[3]
	ADV-R: GCCCCAGTGGTCTTACATGCACATC	400			
	ADV-FAM: TGCACCAGACCCGGGCTCAGGTACTCCGA	200			
RSV	RSV-F: GCAAATATGGAAACATACGTGAACA	500	M gene	MN365598.1 (RSV B) MZ516142.1 (RSV A)	
	RSV-R: GCACCCATATTGTWAGTGATGCA	500			
	RSV-FAM: CTTCACGAAGGCTCCACATACACAGCWG	250			
hRV	HRV-F: GGTGTGAAGAGCCSCRTGTGCT	300	5' UTR	MZ835617.1	
	HRV-F: GGTGTGAAGACTCGCATGTGCT	300			
	HRV-F: GGGTGYGAAGAGYCTANTGTTC	300			
	HRV-R: GGACACCCAAAGTAGTYGGTYC	300			
	HRV-FAM: CCGGCCCTGAATGYGGCTAAYC	250			
MPV	MPV-F: CAGCAATGTCTGTACTTC	500	Matrix protein gene	MN745087.1	
	MPV-R: GACTGTGAGTTTGTCAAA	500			
	MPV-CY5: TCAATGCGACTGTAGC	250			
hBoV	hBoV-F: CAAATCTCTTCTGGCTACACG	500	NS1 gene	KY629422.1	
	hBoV-R: CTCTGCGATCTCTATATTGAAGG	500			
	hBoV-FAM: ATGTTGCCGCCAGTAACTCCACC	250			
hPIV 1	hPIV1-F: ACCTACAAGGCAACAACATC	400	HN gene	MW654396.1	
	hPIV1-R: CTTCCTGCTGGTGGTTAAT	400			

	hPIV1-FAM: TTGGTCTACAACCCGAAATGATAACTCCACGG	250		
hPIV 2	hPIV2-F: GGAGATTGCCTCGATTTCACGAC	400	HN gene	KT898922.1
	hPIV2-R: GTCTCAGTTCAGCTAGATCAG	400		
	hPIV2-VIC: TCTGCTGCAGGGTTTCCAATTTTCAGGACT	250		
hPIV 3	hPIV3-F: GGAGCATTGTCATCTTGTC	400	HN gene	M21649.1
	hPIV3-R: TAGTGTGTAATGCAGCTCGT	400		
	hPIV3-CY5: TGTTTCGGATGGCCAGCTCGTTTACTC	250		

REFERENCES

1. Zhang H, Wang Y, Porter E, et al. Development of a multiplex real-time RT-PCR assay for simultaneous detection and differentiation of influenza A, B, C, and D viruses. Diagn Microbiol Infect Dis. 2019; 95(1):59–66.
2. CDC. Research Use Only CDC Flu SC2 Multiplex Assay Primers and Probes [Internet]. 2021 [cited 2021 Nov 12]. Available from: https://www.cdc.gov/coronavirus/2019-ncov/lab/multiplex-primer-probes.html
3. Gunson RN, Maclean AR, Shepherd SJ, Carman WF. Simultaneous detection and quantitation of cytomegalovirus, Epstein-Barr virus, and adenovirus by use of real-time PCR and pooled standards. J Clin Microbiol. 2009; 47(3):765–770.
