# Supplementary material for: Nasopharyngeal carriage of Streptococcus pneumoniae among Brazilian children: Interplay with viral co-infection
Source: PLoS One. 2025 Jan 2;20(1):e0316444. doi: 10.1371/journal.pone.0316444 (PMC11694996; doi:10.1371/journal.pone.0316444)
Supplement: S2 Table — (PDF) [file pone.0316444.s002.pdf]

S2 Table. Socio-demographic factors associated with Only Pneumococcal carriage, Only Respiratory viruses detection, Co-occurrence in 229 children, Veranópolis, Brazil, 2018-2019.

**Socio-demographic factors**	**Microorganisms**		***p-value***	***OR***
	Only Pneumococcal carriage, n = 66	No agents identified, n=50		
Age months, median [IQR]	42 [30; 51]	42 [29; 54]	0.822	1.00 (0.97; 1.03)
Male Sex	30 (45.5%)	17 (34%)	0.166	1.73 (0.80; 3.78)
Doesn't sleep alone	41 (62.1%)	35 (70%)	0.379	1.44 (0.64; 3.21)
Spring	9 (13%)	8 (16%)		1.00
Summer	2 (3%)	2 (4%)	0.697	0.64 (0.07; 5.98)
Fall	30 (45.5%)	17 (34%)	0.487	1.50 (0.48; 4.65)
Winter	25 (37.9%)	23 (46%)	0.803	0.87 (0.28; 2.68)
Any illness symptoms at the interview	42 (63.6%)	19 (38%)	0.766	0.89 (0.41; 1.93)
	Only Respiratory viruses detection, n = 33	No agents identified, n=50		
Age in Months, median [IQR]	31 [25; 47]	42 [29; 54]	**0.020**	**0.96 (0.92; 0.99)**
Male Sex	17 (51.5%)	17 (34%)	0.161	1.95 (0.77; 4.98)
Doesn't sleep alone	27 (81.8%)	35 (70%)	0.242	0.52 (0.17; 1.56)
Spring	3 (9.1%)	8 (16%)		1.00
Summer	1 (3%)	2 (4%)	0.861	1.29 (0.07; 22.38)
Fall	10 (30.3%)	17 (34%)	0.520	1.68 (0.35; 8.08)
Winter	19 (57.6%)	23 (46%)	0.323	2.14 (0.47; 9.64)
Any illness symptoms at the interview	18 (54.5%)	19 (38%)	0.342	1.57 (0.62; 3.97)
	Co-occurrence, n = 80	No agents identified, n=50		
Age in Months, median [IQR]	33 [24; 43]	42 [29; 54]	**0.003**	**0.95 (0.92; 0.98)**
Male Sex	51 (63.8%)	17 (34%)	**0.001**	**3.62 (1.64; 7.98)**
Doesn't sleep alone	60 (75%)	35 (70%)	0.583	0.79 (0.34; 1.85)
Spring	4 (5%)	8 (16%)		1.00
Summer	1 (1.3%)	2 (4%)	0.796	0.69 (0.04; 11.19)
Fall	45 (56.3%)	17 (34%)	**0.018**	**5.3 (1.33; 21.09)**
Winter	30 (37.5%)	23 (46%)	0.252	2.25 (0.56; 8.98)
Any illness symptoms at the interview	40 (50%)	19 (38%)	0.191	1.68 (0.77; 3.66)

OR: Odds Ratio; Multinomial Logistic Regression

p < 0.05 significant
