# Supplementary material for: Nasopharyngeal carriage of Streptococcus pneumoniae among Brazilian children: Interplay with viral co-infection
Source: PLoS One. 2025 Jan 2;20(1):e0316444. doi: 10.1371/journal.pone.0316444 (PMC11694996; doi:10.1371/journal.pone.0316444)
Supplement: S3 Table — (PDF) [file pone.0316444.s003.pdf]

S3 Table. Frequency of respiratory viruses in children with and without pneumococcal carriage, Veranópolis/RS, Brazil, between 2018 and 2019.

		Pneumococcal carriage		
	**Respiratory virus detection**	**Yes (n=146)**	**No (n=83)**	***p-value***
**hRV**	Alone (n = 51)	38 (26%)	13 (15.7%)	0.100
	Total[1] (n = 75)	57 (39%)	18 (21.7%)	**0.011**
	Virus Co-occurrence (n = 24)			
	hRV + BOV (n = 1)			
	hRV + ADV (n = 4)			
	hRV + ADV + BOV (n = 2)			
	hRV + ADV + MPV (n = 2)			
	hRV + ADV + RSV (n = 1)			
	hRV + MPV (n = 5)			
	hRV + MPV + BOV (n = 4)			
	hRV + RSV (n = 2)			
	hRV + RSV + MPV (n = 1)			
	hRV + RSV + BOV (n = 1)			
	hRV + ADV + MPV + RSV (n = 1)			
**ADV**	Alone (n = 9)	6 (4.1%)	3 (3.6%)	1.000
	Total[1] (n = 23)	18 (12.3%)	5 (6%)	0.195
	Virus Co-occurrence (n = 14)			
	hRV + ADV (n = 4)			
	hRV + ADV + BOV (n = 2)			
	hRV + ADV + MPV (n = 2)			
	hRV + ADV + RSV (n = 1)			
	hRV + ADV + MPV + RSV (n = 1)			
	ADV + BOV (n = 2)			
	ADV + BOV + RSV (n = 1)			
	ADV + BOV + RSV + MPV (n = 1)			

**hBOV**	Alone (n = 13)	4 (2.7%)	9 (10.8%)	**0.016**
	Total[1] (n = 27)	16 (11%)	11 (13.3%)	0.761
	Virus Co-occurrence (n = 14)			
	hRV + BOV (n = 1)			
	hRV + ADV + BOV (n = 2)			
	hRV + MPV + BOV (n = 4)			
	hRV + RSV + BOV (n = 1)			
	ADV + BOV (n = 2)			
	ADV + BOV + RSV (n = 1)			
	ADV + BOV + RSV + MPV (n = 1)			
	BOV + MPV (n = 1)			
	BOV + RSV (n = 1)			
**RSV**	Alone (n = 8)	7 (4.8%)	1(1.2%)	0.264
	Total[1] (n = 17)	14 (9.6%)	3 (3.6%)	0.163
	Virus Co-occurrence (n = 9)			
	hRV + RSV (n = 2)			
	hRV + RSV + MPV (n = 1)			
	hRV + RSV + BOV (n = 1)			
	hRV + ADV + RSV (n = 1)			
	hRV + ADV + MPV + RSV (n = 1)			
	ADV + BOV + RSV (n = 1)			
	ADV + BOV + RSV + MPV (n = 1)			
	BOV + RSV (n = 1)			
**MPV**	Alone (n = 2)	1 (0.7%)	1 (1.2%)	1.000
	Total[1] (n = 17)	13 (8.9%)	4 (4.8%)	0.384
	Virus Co-occurrence (n = 15)			
	hRV + ADV + MPV (n = 2)			
	hRV + MPV (n = 5)			

hRV + MPV + BOV (n = 4)			
hRV + RSV + MPV (n = 1)			
hRV + ADV + MPV + RSV (n = 1)			
ADV + BOV + RSV + MPV (n = 1)			
BOV + MPV (n = 1)			
**Any respiratory virus (n=113)**	80 (54.8%)	33 (39.7%)	**0.040**

The chi-square or Fisher's exact test

p < 0.05 significant

[1] Total = alone + co-detection with another respiratory virus
