# Supplementary material for: Nasopharyngeal carriage of Streptococcus pneumoniae among Brazilian children: Interplay with viral co-infection
Source: PLoS One. 2025 Jan 2;20(1):e0316444. doi: 10.1371/journal.pone.0316444 (PMC11694996; doi:10.1371/journal.pone.0316444)
Supplement: S4 Table — (PDF) [file pone.0316444.s004.pdf]

S4 Table. Frequency of clinical symptoms and diagnosis in medical care within 14 days according to different respiratory viruses, Veranópolis/RS, Brazil, between 2018 and 2019.

	Total	hRV	ADV	hBOV	RSV	MPV	Any respiratory virus
Medical records n, (%)	224	74 (33%)	22 (9.8%)	27 (12.1%)	17 (7.6%)	17 (7.6%)	111 (49.6%)
Medical care within 14 days, n	41 (18.3%)	13 (17.6%)	3 (3.6%)	7 (25.9%)	4 (23.5%)	9 (52.9%)	21 (18.9%)
Cough	24 (10.7%)	6 (8.1%)	2 (9.1%)	4 (14.8%)	2 (11.8%)	6 (35.3%)	11 (9.9%)
Nasal congestion/ coryza	18 (8.0%)	4 (5.4%)	2 (9.1%)	1 (3.7%)	-	5 (29.4%)	6 (5.4%)
Fever	13 (5.8%)	3 (4.1%)	1 (4.5%)	-	1 (5.9%)	4 (23.5%)	4 (3.6%)
Sore throat	8 (3.6%)	3 (4.1%)	1 (4.5%)	2 (7.4%)	-	1 (5.9%)	4 (3.6%)

The chi-square or Fisher's exact test
