# Supplementary material for: Nasopharyngeal carriage of Streptococcus pneumoniae among Brazilian children: Interplay with viral co-infection
Source: PLoS One. 2025 Jan 2;20(1):e0316444. doi: 10.1371/journal.pone.0316444 (PMC11694996; doi:10.1371/journal.pone.0316444)
Supplement: S5 Table — (PDF) [file pone.0316444.s005.pdf]

S5 Table. Detection of different respiratory viruses according to Seasons, Veranópolis/RS, Brazil, between 2018 and 2019.

	**Summer**	**Fall**	**Winter**	**Spring**	***p-value***
Respiratory viruses, n (%)	6 (2.6%)	102 (44.5%)	97 (42.4%)	24 (10.5%)	17 (7.4%)
hRV	2 (33.3%)	38 (37.3%)	30 (30.9%)	5 (20.8%)	0.451
ADV	0 (0%)	13 (12.7%)	8 (8.2%)	2 (8.3%)	0.590
hBOV	0 (0%)	10 (9.8%)	17 (17.5%)	0 (0%)	0.058
RSV	0 (0%)	**13 (12.7%)**	4 (4.1%)	0 (0%)	**0.043**
MPV	0 (0%)	11 (10.8%)	4 (4.1%)	2 (8.3%)	0.293

The chi-square or Fisher's exact test

$p < 0.05$ significant
