# Supplementary material for: Nasopharyngeal carriage of Streptococcus pneumoniae among Brazilian children: Interplay with viral co-infection
Source: PLoS One. 2025 Jan 2;20(1):e0316444. doi: 10.1371/journal.pone.0316444 (PMC11694996; doi:10.1371/journal.pone.0316444)
Supplement: Attachment — Submitted filename: rebuttal letter.docx [file pone.0316444.s006.docx]

**Additional Editor Comments:**

While your paper addresses an interesting question, the reviewers stated several concerns about the presentation as well as the readability of the manuscript and did not recommend publication in its present form. In particular, reviewers voice a number of concerns regarding the data analysis, and these comments need to be addressed carefully. Please see reviewers’ insightful comments below. Personally, at a more detailed level, I find the authors should take care on how the data was calculated, for example, the prevalence of microorganism detection should based on the final number included (n=224) for analysis not the original number (n=230)

We would like to thank the editors and reviewers for their dedication and thorough analysis of our manuscript. We were able to implement most of the suggestions, and in the new version the number of samples had to be changed due to a technical sample loss (from 230 to 229). As for the editor's request, we can't implement it because the number of 224 is the result of the analysis of medical records (cohort analysis) in which the sample loss was 5 children, which doesn't change the value of the prevalence of the microorganisms in the cross-sectional study of the 229 children. Information included in the Flowchart on line 157

In addition, the quality of the language needs to be improved. Please have a fluent, preferably native, English-language speaker thoroughly copyedit your manuscript for language usage, spelling, and grammar.

The English has been revised as requested. In addition to other requests from the reviewers, lines and some references have been changed.

Reviewer #1: The authors present an interesting investigation into pneumococcal carriage and detection of respiratory viruses.

We would like to thank the reviewer for his dedication and thorough analysis of our manuscript. We were able to improve it a lot with your support

Abstract:
Line 26: I suggest something like ‘prerequisite’ rather than ‘compulsory’

It was modified as suggested.

Lines 27-28: This sentence starts by discussing the relationship between carriage and infection, but then goes on to say that the study includes asymptomatic children – which is indicative of lack of infection. This should be rephrased for clarity

The sentence was rephrased as suggested, line 29: "in healthy children from the general population in southern Brazil"

Lines 42-43: from the text in the abstract, it is not clear how this work contributes to the understanding ‘between these agents and their implications for disease’ – there is no evidence of associations with virus or co-infection and outcomes?

The text was reviewed and modified. Line 46: "potentially pathogenic agents."

Methods:
Line 91: What determines a ‘satisfactory’ NP swab?

This sentence was rephrased, the collection guideline was included. Line 90: "Participants were recruited as volunteers through schools, telephone calls, and radio announcements in the city. All legal guardians provided written informed consent signed at the moment of specimen collection using the informed consent form (ICF). If siblings were included, the legal guardian provided consent for both children. All participants were considered healthy enough to attend school and maintain a normal routine at the time of collection. Individuals were deemed ineligible for enrollment if they had been diagnosed with acute respiratory disease or had been hospitalized in the week preceding specimen collection. Health professionals trained for the study conducted structured interviews with legal guardians to collect data on demographic characteristics, pneumococcal vaccination history, and the presence of symptoms such as cough, coryza/congestion, or sore throat in the child. Children did not undergo medical examinations."

Line 111: superscript the ‘TM’ on the magmax kit (it is the trademark symbol, not an abbreviation of the kit itself).
The alteration was done. line 121.

Results:
The start of this section would benefit from a little more introduction of the enrolled study population characteristics.

The text was reviewed and modified. Line 144: "In 2017, Veranópolis City had an estimated 781 children aged 18–59 months."

Line 155: ‘females’ instead of ‘girls.

It was modified as suggested. line 147.

Line 188: seasonality can be tested/controlled for in a multivariate analysis – this should be checked – as should they other demographic features as well as pathogen detection. Here, you can then also look at household size, school attendance etc,

The suggestion was accepted and Table 1 included all the data from the questionnaire, including seasonality. The multivariate data is in the narrative text and table 2 of the supplementary material. line 167: "Additionally, a multinomial logistic regression analysis of certain demographic data and the presence of only pneumococcal carriage, respiratory virus, and co-occurrence was conducted (S2 Table)."

Table 1: it is currently not clear, when reporting significance, what the comparator is. A more robust statistical analysis of this data is required and would strength the data in the field on the associations between demographics and pathogen detection as well as the interplay between pneumococcus and viruses within these groups.

In the multivariate (new S2 Table 1) each outcome in relation to "None", male sex in relation to female, absence of symptoms in relation to presence and each season in relation to spring.

In table 1 there is no comparator: it is the association of the variable with the microorganism variable. we have included the youngest age in the footnote of table 1

It was modified as suggested. "⁴ Children who tested positive for virus infection and co-occurrence were younger than those that were pneumococcal carriers only"

Discussion:
Did the study team record the density of pneumococcus growth on culture plate? This could be used to look for an association between density and virus type.

Unfortunately this has not been reported as it was grown in broth and then seeded in culture

The paper would benefit from more discussion of hBOV since this is a more novel finding – the discussion on HRV could be condensed – the different studies could be summarized more succinctly.

We understand but we chose to keep a longer discussion with hRV because now we have brought new results and there has been an association with the symptom of sore throat (line 210). A new text on bocavirus has been reformulated to emphasize the "novel finding" (line 247)

Line 210: "Children who were "too sick" were excluded from this study. The recruitment process allowed for the inclusion of children with mild respiratory symptoms, as reported by their guardians. Consequently, 108 (47.0%) and 79 (34.5%) of the children exhibited coryza/congestion and cough, respectively. Despite the frequency of these findings in our study, they were not associated with the detection of the studied viruses. In the literature, these symptoms are commonly associated with respiratory viruses. Notably, lower levels of Ct are often associated with greater severity of respiratory symptoms and mortality [40]. However, in our study, high Ct levels were observed, aligning with the clinical condition of the study group (considered healthy) and the lack of association with symptoms. Also, in five of the children, guardians reported sore throat, and in four of them, hRV was detected. Statistically significant associations were observed between any level of hRV Ct values (p<0.05) and sore throat, rather than only low hRV Ct values. Some studies have demonstrated the association of hRV with sore throat [41,42]. For instance, in a study conducted with individuals experiencing sore throat, hRV was the most commonly detected pathogen [43]. Moreover, an experimental study involving intranasal inoculation of hRV in humans observed scratchy/sore throat symptoms appearing 10 to 12 hours after induced inoculation [44]. It's important to note that as these observations about symptoms were not derived from a medical examination, some subjectivity is involved. In our study, INF and PIV were not found. The prevalence of these viruses is typically low (<3%) among asymptomatic children [15,16], as they are known to interact with hosts, producing symptoms [45,46]."

line 247: "Similarly, a Japanese study observed a positive correlation between the co-detection of pneumococcus and hBOV, where 85.7% (12/14) of children tested positive for both pathogens in otitis media fluid (MEF). However, in the nasopharynx, this correlation was not observed, as the proportion of pneumococcus was similar between groups of individuals positive and negative for hBOV. Notably, pneumococcus was the only pathogen present in all but one of the co-detections in hBOV-positive cases, whereas in the nasopharynx, it was mixed with other pathogenic bacteria [53]. This suggests that these pathogens migrate to their target sites, with the nasopharynx not being the site where the etiologic agent is identified It is essential to highlight that in this site (the nasopharynx), children without serious disease may act as antagonists, potentially suggesting protection of pneumococcus in the absence of hBOV. However, when present together, this migration may be favored. One common weakness of these studies is the small sample size, which can hinder drawing definitive conclusions about the relationship between hBOV and pneumococcus. Nevertheless, these studies collectively underscore the relevance of pneumococcus and respiratory virus co-infection in disease pathogenesis. Our study's findings, such as the absence of INF and PIV in the sampled population, likely reflect the predominance of these viruses in children with more severe symptoms. INF, in particular, is well-known for its relationship with co-infection and increased risk of subsequent severe pneumococcal disease. Further studies are needed to elucidate the mechanisms underlying the interaction between hBOV and pneumococcus."

It could be useful to report the Ct values of the virus qPCR detection – if they are low viral loads (high Ct values) this would suggest either a resolving or historic infection (which I commend the authors for recognizing in the text!) so less clinically relevant. If any are higher viral loads (lower Ct) this would suggest more active infection and would be more interesting.

It was modified as suggested. A new table 2 has been included with virus ct analysis. Line 177:

| **Table 2. Frequency of clinical symptoms in children with detection of respiratory viruses by real-time PCR using the cut-off levels Cycle threshold (CT), Ct- value of < 30 (only high levels) and < 35 (any level), Veranópolis/RS, Brazil, between 2018 and 2019.** | | | | | | | | | | | | | |
| --- | --- | --- | --- | --- | --- | --- | --- | --- | --- | --- | --- | --- | --- |
|  | Total | RNV | | ADV | | BOV | | RSV | | MPV | | Any respiratory virus | |
| Cycle Threshold (CT) |  | <30 | <35 | <30 | <35 | <30 | <35 | <30 | <35 | <30 | <35 | <30 | <35 |
| n, (%) | 229 | 33 (14.4) | 75 (32.8) | 9 (3.9) | 23 (10) | 6 (2.6) | 27 (11.8) | 3 (1.3) | 17 (7.4) | 2 (0.9) | 17 (7.4) | 47 (20.5) | 113 (49.3) |
| Cough | 79 (34.5) | 11 (33.3) | 26 (34.7) | 2 (22.2) | 5 (21.7) | 0  (0) | 8 (29.6) | 0  (0) | 4 (23.5) | 0  (0) | 5 (29.4) | 13 (27.7) | 38  (33.6) |
| Nasal congestion/coryza | 108 (47.2) | 17 (51.5) | 35 (46.7) | 3 (33.3) | 10 (43.5) | 1 (16.7) | 10 (37) | 0  (0) | 5 (29.4) | 1 (50) | 7 (41.2) | 21 (44.7) | 48  (42.5) |
| Sore throat | 5 (2.2) | 2  (6.1) | 4 (5.3) * | 0  (0) | 0  (0) | 0  (0) | 0  (0) | 0  (0) | 0  (0) | 0  (0) | 0  (0) | 2 (4.3) | 4  (3.5) |
| The chi-square or Fisher’s exact test | | | | | | | | | | | | | |
| *p < 0.05 significant | | | |  | | | |  |  |  |  |  |  |

Reviewer #2: Major

We would like to thank the reviewer for his dedication and thorough analysis of our manuscript. We were able to improve it a lot with your support

- The manuscript needs to be reviewed for English language.

We carry out a new english revision

- The data statement indicates that data are available within the manuscript but only aggregate are provided and not individual-level data.

We have added an excel file to include all the results obtained for each individual child, new Supplementary Information 3

- Study design – this is a cohort study with a cross-sectional analysis and cohort analysis. It should not be described as a cross-sectional cohort study.

It was modified as suggested. line 86:"We conducted a cross-sectional cohort study to investigate pneumococcal carriage and respiratory viruses in the nasopharynx of children aged 18–59 months."

- Methods – more details are needed to understand who is in the study and interpret the results:
o It looks like all children in the city were eligible – how were they recruited and were siblings allowed/enrolled. If siblings were allowed, then how was that accounted for in the analysis?

The recruitment was by convenience sampling. line 90: "Participants were recruited as volunteers through schools, telephone calls, and radio announcements in the city."

Siblings were included. line 92: "if siblings were included, the legal guardian provided consent for both children."

(it’s not clear in Table 1 if ‘more than one child in the same household’ refers to enrolled in the study or just present)

we don't exclude children from being in the same house (see inclusion and exclusion criteria). Line: "Participants were recruited as volunteers through schools, telephone calls, and radio announcements in the city. All legal guardians provided written informed consent signed at the moment of specimen collection using the informed consent form (ICF). If siblings were included, the legal guardian provided consent for both children."

o The children were considered ‘healthy’ and are called ‘asymptomatic’ throughout the manuscript. They were excluded if they had been diagnosed with ARI or hospitalized the week before. It does not appear that they were excluded if they had respiratory symptoms before (not medically attended) or at the visit. If they were excluded then this should be stated. If they were not excluded, then it should be reported in table 1 how many were symptomatic before or at the visit and the participants should not be referred to as ‘asymptomatic’.

Was accepted and changed from "asymptomatic" to "healthy". As described in line 93: "All participants were considered healthy enough to attend school and maintain a normal routine at the time of collection. Individuals were deemed ineligible for enrollment if they had been diagnosed with acute respiratory disease or had been hospitalized in the week preceding specimen collection. Health professionals trained for the study conducted structured interviews with legal guardians to collect data on demographic characteristics, pneumococcal vaccination history, and the presence of symptoms such as cough, coryza/congestion, or sore throat in the child. Children did not undergo medical examinations."

I would also remove them from the cohort analysis since they already have symptoms or model incidence to account for this.

the 14-day analysis was to analyze whether seeking care was an aggravation of the symptoms or a possible diagnosis (of interest to the study)

o The cohort analysis relies on medical records for the outcome. The authors state that they checked 15 different health systems, which seems comprehensive but without knowing this area it is hard to understand if this represents all of the health systems that they could have checked or only a subset. If it’s only a subset, then the completeness of the outcome ascertainment is called into question and it’s not clear if the lack of association is due to misclassification.

The text was reviewed and modified. Line: 103: "covering the entire city"
o The cohort analysis is a univariable analysis. Are there other important factors that affect healthcare seeking (e.g., SES) that may have differed between groups that should be accounted for? As few variables are presented in Table 1, it is hard to know.

It was modified as suggested. A more complete table 1 (line 166) with all the data from the questionnaire and we carried out multivariate analysis (included a new table: S2 Table 1 in Supplementary Information) only for the statistically significant data from the univariate analysis.

| **Table 1. Demographic characteristics of the population (n = 229) according to identified microorganisms, Veranópolis/RS, Brazil, 2018–2019** | | | | | | |
| --- | --- | --- | --- | --- | --- | --- |
|  | Total | Only Pneumococcal carriage | Only Respiratory viruses detection | Co-occurrence ³ | No agents identified | *p-value* |
| **n, (%)** | 229 | 66 (28.8%) | 33 (14.4%) | 80 (34.9%) | 50 (21.8%) |  |
| **Age months, median [IQR]** | 36 [27; 48] | 42 [30; 51] | 31 [25; 47] | 33 [24; 43] | 42 [29; 54] | **<0.001*⁴** |
| **Male Sex** | 115 (50.2%) | 30 (45.5%) | 17 (51.5%) | 51 (63.8%) | 17 (34%) | **0.008*** |
| **Self-report ethnicity** |  |  |  |  |  | 0.428 |
| White | 212 (92.5%) | 58 (87.9%) | 31 (93.9%) | 77 (96.3%) | 46 (92%) |  |
| Black | 2 (0.9%) | 0 (0%) | 0 (0%) | 1 (1.3%) | 1 (2%) |  |
| Mixed race | 13 (5.7%) | 7 (10.6%) | 1 (3%) | 2 (2.5%) | 3 (6%) |  |
| Yellow/Indian | 1 (0.4%) | 1 (1.5%) | 0 (0%) | 0 (0%) | 0 (0%) |  |
| Doesn't know | 1 (0.4%) | 0 (0%) | 1 (3%) | 0 (0%) | 0 (0%) |  |
| **Asthma** | 13 (5.7%) | 3 (4.5%) | 3 (9.1%) | 4 (5%) | 3 (6%) | 0.812 |
| **Diabetes** | 0 (0%) | 0 (0%) | 0 (0%) | 0 (0%) | 0 (0%) | - |
| **Kidney Disease** | 2 (0.9%) | 0 (0%) | 0 (0%) | 1 (1.3%) | 1 (2%) | 0.629 |
| **Heart Disease** | 1 (0.4%) | 0 (0%) | 0 (0%) | 0 (0%) | 1 (2%) | 0.309 |
| **Liver Disease** | 1 (0.4%) | 0 (0%) | 0 (0%) | 0 (0%) | 0 (0%) | 0.600 |
| **Cystic Fibrosis** | 0 (0%) | 0 (0%) | 0 (0%) | 0 (0%) | 0 (0%) | - |
| **Sickle cell Anemia** | 2 (0.9%) | 1 (1.5%) | 0 (0%) | 1 (1.3%) | 0 (0%) | 0.759 |
| **Cancer** | 0 (0%) | 0 (0%) | 0 (0%) | 0 (0%) | 0 (0%) | - |
| **Vaccine status ¹** | 225 (98.3%) | 65 (98.5%) | 31 (93.9%) | 80 (100%) | 49 (98%) | 0.169 |
| **School attendance** | 213 (93%) | 61 (92.4%) | 31 (93.9%) | 77 (96.3%) | 44 (88%) | 0.347 |
| **More than one child < 5 years old lives in the same household** | 32 (14%) | 6 (9.1%) | 7 (21.2%) | 11 (13.8%) | 8 (16%) | 0.404 |
| **Other children< 5 years old sleep in the same bedroom of the participant** | 25 (10.9%) | 9 (13.6%) | 6 (18.2%) | 5 (6.3%) | 5 (10%) | 0.248 |
| **Other people sleep in the same bedroom of the participant** | 163 (71.2%) | 41 (62.1%) | 27 (81.8%) | 60 (75%) | 35 (70%) | 0.167 |
| **Family has a member smoker** | 46 (20.1%) | 10 (15.2%) | 10 (30.3%) | 18 (22.5%) | 8 (16%) | 0.266 |
| **Education levels of legal guardians** |  |  |  |  |  | 0.801 |
| Elementary school | 38 (16.6%) | 13 (19.7%) | 7 (21.2%) | 12 (15%) | 6 (12%) |  |
| High school | 80 (34.9%) | 19 (28.8%) | 11 (33.3%) | 31 (38.8%) | 19 (38%) |  |
| Undergraduate/ graduate | 111 (48.5%) | 34 (51.53%) | 15 (45.5%) | 37 (46.3%) | 25 (50%) |  |
| **Breastfeed** |  |  |  |  |  |  |
| Yes | 215 (93.9%) | 61 (92.4%) | 30 (90.9%) | 76 (95%) | 48 (98%) | 0.488 |
| Information unavailable | 1 (0.4%) | 0 (0%) | 0 (0%) | 0 (0%) | 1 (2%) |  |
| **Breastfeeding at the time of interview (n=212) ²** | 32 (15.1%) | 6 (10%) | 7 (23.3%) | 12 (16%) | 7 (14.9%) | 0.415 |
| **Any illness symptoms at the interview** | 131 (57.2%) | 42 (63.6%) | 18 (54.5%) | 40 (50%) | 31 (62%) | 0.337 |
| Cough | 79 (34.5%) | 22 (33.3%) | 12 (36.4%) | 26 (32.5%) | 19 (38%) | 0.918 |
| Nasal congestion/coryza | 108 (47.2%) | 37 (56.1%) | 14 (42.4%) | 34 (42.5%) | 23 (46%) | 0.347 |
| Sore throat | 5 (2.2%) | 0 (0%) | 0 (0%) | 4 (5%) | 1 (2%) | 0.158 |
| **Seasons of the year of sample collection** |  |  |  |  |  | 0.146 |
| Summer | 6 (2.6%) | 2 (3%) | 1 (3%) | 1 (1.3%) | 2 (4%) |  |
| Fall | 102 (44.5%) | 30 (45.5%) | 10 (30.3%) | 45 (56.3%) | 17 (34%) |  |
| Winter | 97 (42.4%) | 25 (37.9%) | 19 (57.6%) | 30 (37.5%) | 23 (46%) |  |
| Spring | 24 (10.5%) | 9 (13.6%) | 3 (9.1%) | 4 (5%) | 8 (16%) |  |
| Age: Kruskal-Wallis + Bonferroni test; other variables: the chi-square | | | | | | |
| *** p< 0.05 significant.** | | | | | | |
| ¹ At least one dose PCV 10 and/or PCV13 vaccination. | | | | | | |
| ² 17 legal guardian didn't answer if the child was still being breastfeeding | | | | | | |
| ³ Co-occurrence = Pneumococcal carriage and respiratory virus detection.  ⁴ Children who tested positive for virus infection and co-occurrence were younger than those that were pneumococcal carriers only | | | | | | |

**S2 Table 1. Socio-demographic factors associated with Only Pneumococcal carriage, Only Respiratory viruses detection, Co-occurrence in 229 children, Veranópolis, Brazil, 2018-2019**

| **Socio-demographic factors** | **Microorganisms** | ***p-value*** | ***OR*** |
| --- | --- | --- | --- |
|  | Only Pneumococcal carriage, n = 66 |  |  |
| Age in Months | 42 [30; 51] | 0,822 | 1.00 (0.97; 1.03) |
|  |  |  |  |
| Male Sex | 30 (45.5%) | 0,166 | 1.73 (0.80; 3.78) |
| Doesn't sleep alone | 41 (62.1%) | 0,379 | 1.44 (0.64; 3.21) |
| Summer | 2 (3%) | 0,697 | 0.64 (0.07; 5.98) |
| Fall | 30 (45.5%) | 0,487 | 1.50 (0.48; 4.65) |
| Winter | 25 (37.9%) | 0,803 | 0.87 (0.28; 2.68) |
| Any illness symptoms at the interview | 42 (63.6%) | 0,766 | 0.89 (0.41; 1.93) |
|  | Only Respiratory viruses detection, n = 33 |  |  |
| Age in Months | 31 [25; 47] | **0,020*** | **0.96 (0.92; 0.99)*** |
| Male Sex | 17 (51.5%) | 0,161 | 1.95 (0.77; 4.98) |
| doesn't sleep alone | 27 (81.8%) | 0,242 | 0.52 (0.17; 1.56) |
| Summer | 1 (3%) | 0,861 | 1.29 (0.07; 22.38) |
| Fall | 10 (30.3%) | 0,520 | 1.68 (0.35; 8.08) |
| Winter | 19 (57.6%) | 0,323 | 2.14 (0.47; 9.64) |
| Any illness symptoms at the interview | 18 (54.5%) | 0,342 | 1.57 (0.62; 3.97) |
|  | Co-occurrence,  n = 80 |  |  |
| Age in Months | 33 [24; 43] | **0,003*** | **0.95 (0.92; 0.98)*** |
| Male Sex | 51 (63.8%) | **0,001*** | **3.62 (1.64; 7.98)*** |
| doesn't sleep alone | 60 (75%) | 0,583 | 0.79 (0.34; 1.85) |
| Spring |  |  | 1,00 |
| Summer | 1 (1.3%) | 0,796 | 0.69 (0.04; 11.19) |
| Fall | 45 (56.3%) | **0,018*** | **5.3 (1.33; 21.09)*** |
| Winter | 30 (37.5%) | 0,252 | 2.25 (0.56; 8.98) |
| Any illness symptoms at the interview | 40 (50%) | 0,191 | 1.68 (0.77; 3.66) |
| OR: Odds Ratio; Multinomial Logistic Regression  ***p < 0.05 significant** | | | |

o Respiratory sample collection and testing – provide brief methods (e.g., broth enrichment) so the reader can know what was done in this study without reviewing another protocol.

The text was reviewed and modified. Line 118: " involving broth enrichment followed by growth on blood agar base."

o Statistical analysis – the authors state that the sample size was determined based on a desired confidence interval. However, no confidence intervals are presented. Is there another paper from this study that should be cited in the Methods where the primary outcome (prevalence of PNC) is reported?

In the static item there was already the reference value line: 132 "The sample size was calculated considering a PNC prevalence of 62.3% [36]" and your sample had "63.7% (95%CI: 57.4 - 69.8)" in line 159.

o Discussion:
 Paragraph starting on line 201: Suggest refocusing to discuss that the study is consistent about an association between PNC and viruses. There was an association with hRV but it was also the most common virus detected. Similar trends were observed for all other viruses except hBOV, but with smaller sample sizes which likely contributed to the lack of statistical significance.

Thank you for the suggestion and we have already included the most relevant results in the first paragraph by discussion. As for the association of hRV and the significant association, no similar trend was found in other viruses.

Paragraph starting online 219: since this is the major finding, I suggest expanding the discussion unpack a little more what you measured for both exposure and outcome.

We included a longer text about how hbov and ct were further explored in the discussion.

line 247: "Similarly, a Japanese study observed a positive correlation between the co-detection of pneumococcus and hBOV, where 85.7% (12/14) of children tested positive for both pathogens in otitis media fluid (MEF). However, in the nasopharynx, this correlation was not observed, as the proportion of pneumococcus was similar between groups of individuals positive and negative for hBOV. Notably, pneumococcus was the only pathogen present in all but one of the co-detections in hBOV-positive cases, whereas in the nasopharynx, it was mixed with other pathogenic bacteria [53]. This suggests that these pathogens migrate to their target sites, with the nasopharynx not being the site where the etiologic agent is identified It is essential to highlight that in this site (the nasopharynx), children without serious disease may act as antagonists, potentially suggesting protection of pneumococcus in the absence of hBOV. However, when present together, this migration may be favored. One common weakness of these studies is the small sample size, which can hinder drawing definitive conclusions about the relationship between hBOV and pneumococcus. Nevertheless, these studies collectively underscore the relevance of pneumococcus and respiratory virus co-infection in disease pathogenesis. Our study's findings, such as the absence of INF and PIV in the sampled population, likely reflect the predominance of these viruses in children with more severe symptoms. INF, in particular, is well-known for its relationship with co-infection and increased risk of subsequent severe pneumococcal disease. Further studies are needed to elucidate the mechanisms underlying the interaction between hBOV and pneumococcus."

• You only measured a subset of viruses and did not measure some major respiratory viruses (e.g., influenza, seasonal coronaviruses)

The influenza and PIV viruses were analyzed and did not have a positive sample. line 181: "All samples tested negative for influenza A and B, as well as parainfluenza viruses 1, 2, and 3. "

We discuss the low incidences in outgoing children in other studies in line 226 "In our study, INF and PIV were not found. The prevalence of these viruses is typically low (<3%) among asymptomatic children [15,16], as they are known to interact with hosts, producing symptoms [45,46]."

In line 287 Includes limitations related to the non-inclusion of seasonal coronaviruses: "Furthermore, our viral panel, while comprehensive, did not include other agents that could potentially contribute to pneumococcal colonization, such as seasonal coronaviruses."

• It’s also not clear with PCR what you measured given that children were 'asymptomatic' – did you measure past infection or did you measure recent infection and the healthcare visits in the next 14 days were for the respiratory viral infection? Was this the right window for detecting pneumococcal infection after a viral infection?

The text was reviewed and modified. Line 101: "This period was selected because the respiratory disease and/or symptoms following co-infection between pneumococcus and respiratory viruses appear to act within a period of 1 to 2 weeks [15,16,20-22]."

• The outcome was medically attended visits and not just respiratory symptoms, which indicates a level of severity of the infection.

The text was reviewed and modified. line 265: "We focused on medical consultations, but exclusively recorded symptoms and diagnoses of interest for this study."

Minor
- Throughout the manuscript be consistent in the number of decimals presented and how numbers are presented (period vs. comma).

It was modified as suggested.
- Introduction, line 75: the authors state that they considered the increase in infectious disease severity when pathogens interact. This should be rephrased to more accurately reflect what was done.

It was modified as suggested. line 79: "Several studies have explored the heightened severity of infectious diseases when these pathogens interact"
- Methods
o line 92: fix typo – ‘health’ professionals

It was modified as suggested. line 96: "Health professionals"

o Line 95: remove double period

It was modified as suggested.

- Results
o Line 150: suggest starting out by reporting the % with respiratory viruses by PNC status and providing p-value an then going into co-occurrence.

It was modified as suggested. line 160: "Among those with pneumococcal carriage, 54.8% (80/146; p = 0.033) tested positive for respiratory viruses"

o Line 158: ‘hRV alone + co-detection’ = ‘total’ in the table. Suggest using ‘total (alone + co-detection)’ for ease of reading text.

It was modified as suggested. line 179.
o Line 165: suggest starting by reporting the overall % that required medical care within 14 days.

It was modified as suggested. line 185: "19.2% (43/224) required medical care within 14 days."

o Line 165: the authors indicate ‘co-occurrence was the most frequent factor among the 43 children who needed medical care’ but this is not what is shown in the table and incidence of medical care in this group was among the lowest. Please re-phrase.

It was modified as suggested. line 186: "Co-occurrence was present in 32.5% (14/43) of the children who needed medical care."
- Figure 1
o It looks like no children were excluded for having an ARI – is that correct? Seems odd given detection of viruses.

It was modified as suggested. Line 90: " Participants were recruited as volunteers through schools, telephone calls, and radio announcements in the city."

o Box ‘refused to collect or sign ICF’ has an asterix – what is it for?

It was modified as suggested. line 157: "Fig 1."

o Fix typo in box ‘children included’

It was modified as suggested. line 157: "Fig 1."

- Table1
o Header row (culture isolation …) is unnecessary and can be deleted

It was modified as suggested.

o Clarify that PNC and respiratory virus detection columns are ‘only’

It was modified as suggested. line 166: "Only Pneumococcal carriage" and "Only Respiratory viruses detection"

o Footnote 2 – clarify that this is ‘at least one dose’

It was modified as suggested. line 166: " ¹ At least one dose PCV 10 and/or PCV13 vaccination."

o Footnote 4 is not necessary

It was modified as suggested.

o Mean (SD) is presented for age – confirm that age was normally distributed; if not, then median (IQR) would be more appropriate.

It was modified as suggested.

- Table 2
o Title should be changed as it does not only include children with PNC

It was modified as suggested. line 183: "Table 3. Frequency of respiratory viruses in children with and without pneumococcal carriage, Veranópolis/RS, Brazil, between 2018 and 2019."

o Rows need to be aligned

It was modified as suggested.

o Footnotes 2 and 3 are not necessary

It was modified as suggested.

- Table 3
o Suggested title: incidence of medical appointments 14 days after specimen collection among children, by colonization and respiratory virus detection

It was modified as suggested. line 190: "Table 4. Incidence of medical appointments 14 days after specimen collection among children, by S. pneumoniae colonization and respiratory virus detection "
o As you are presenting incidence (row percents), the ‘no’ column is unnecessary (may cause confusion to include as Table 2 is similarly structured and presents column percents)

It was modified as suggested.
- Table 4
o Delete header row (culture isolation…) - it is not necessary

It was modified as suggested.

o Change first section results row to ‘Any documented clinical symptoms within 14 days’

It was modified as suggested.Table 5: "Any documented clinical symptoms within 14 days, n (%)"
o Change second section results row to ‘Any diagnosis within 14 days’

It was modified as suggested. Table 5: "Any diagnosis within 14 days, n (%)"

- S2 Table 1:
o Make it clearer what the top row ‘medical records’ refers to and that you are presenting row percents. The rest of the table presents column percents.

It was modified as suggested. S2 Table 2.

o Suggest changing ’14 days of medical care’ to ‘medical care within 14 days’

It was modified as suggested. line 26: "S2 Table 2. Frequency of clinical symptoms and diagnosis in medical care within 14 days according to different respiratory viruses, Veranópolis/RS, Brazil, between 2018 and 2019."

o For the individual symptoms, the percents in the ‘total’ column don’t match what is reported

It was modified as suggested.

| **S2 Table 2. Frequency of clinical symptoms and diagnosis in medical care within 14 days according to different respiratory viruses, Veranópolis/RS, Brazil, between 2018 and 2019.** | | | | | | | |
| --- | --- | --- | --- | --- | --- | --- | --- |
|  | **Total** | **hRV** | **ADV** | **hBOV** | **RSV** | **MPV** | **Any respiratory virus** |
| Medical records n, (%) | 224 | 74 (33%) | 22 (9.8%) | 27 (12.1%) | 17 (7.6%) | 17 (7.6%) | 111 (49.6%) |
| Medical care within 14 days, n (%) | 41 (18.3%) | 13 (17.6%) | 3 (3.6%) | 7 (25.9%) | 4 (23.5%) | 9 (52.9%) | 21 (18.9%) |
| Cough | 24 (10.71%) | 6 (8.1%) | 2 (9.1%) | 4 (14.8%) | 2 (11.8%) | 6 (35.3%) | 11 (9.9%) |
| Nasal congestion/coryza | 18 (8.04%) | 4 (5.4%) | 2 (9.1%) | 1 (3.7%) | - | 5 (29.4%) | 6 (5.4%) |
| Fever | 13 (5.8%) | 3 (4.1%) | 1 (4.5%) | - | 1 (5.9%) | 4 (23.5%) | 4 (3.6%) |
| Sore throat | 8 (3.6%) | 3 (4.1%) | 1 (4.5%) | 2 (7.4%) | - | 1 (5.9%) | 4 (3.6%) |
| The chi-square or Fisher’s exact test  *p < 0.05 significant | |  |  |  |  |  |  |

in table 4.
- S2 Table 2 – expand the footnote to indicate the test that yielded the p-value and what comparison is being made.

It was modified as suggested. S2 Table 3: "The chi-square or Fisher’s exact test"

Reviewer #3: It is a very interesting article and there are very few articles in Latin America.

We would like to thank the reviewer for his dedication and thorough analysis of our manuscript. We were able to improve it a lot with your support

Some suggestions
Line 150:
It is not clear what is meant by "The prevalence of microorganism detection found in the children was...", it is suggested to use prevalence of respiratory viruses was…

It was modified as suggested. line 159: "The prevalence was pneumococcal carriage 63.7% (146/229) for and respiratory viruses 49.34% (113/229)."

Line 152:
"As shown in Table 1, children who tested positive for viral infection, with or without coexisting pneumococcus, were younger (34 vs. 40 months; p = 0.001)."

It was modified as suggested. line 161: "As shown in Table 1, children who tested positive for virus infection, with or without co-occurrence of pneumococcus, were younger (31 and 34 months vs. 42 months; p < 0.001)."

Not displayed in table 1

The lack or shortage of studies in Latin America could be included in the discussion.

It was modified as suggested. line 202: "This study represents one of the initial investigations in Latin America to explore the relationship between respiratory viruses and pneumococcal carriage in a healthy child population. "

Line 240-243
The high sensitivity of PCR is not a limitation, but the difficulty of interpreting the presence of viruses is.

It was modified as suggested. line 284: "A limitation of our study, common to others, was the use of PCR to detect respiratory viruses in healthy children. Interpretation of results can be challenging, as detection may indicate prolonged shedding of the virus resulting from a previous episode"

Line 225 and 245
There is talk of high vaccination coverage; vaccination coverage could be included in the introduction in Brazil and in the state where the study is carried out

It was modified as suggested. line 59: "Pneumococcal vaccine coverage in Brazil in 2017 was approximately 92%, with rates reaching 93.7% in southern Brazil [8]."
