# Supplementary material for: Nasopharyngeal carriage of Streptococcus pneumoniae among Brazilian children: Interplay with viral co-infection
Source: PLoS One. 2025 Jan 2;20(1):e0316444. doi: 10.1371/journal.pone.0316444 (PMC11694996; doi:10.1371/journal.pone.0316444)
Supplement: Attachment — Submitted filename: Response to Reviewers II.docx [file pone.0316444.s007.docx]

We appreciate all the suggestions of reviewer 1 and 2 that, we are convinced of this, will improve our article. We examined each of the suggestions in detail and did our best to achieve a satisfactory answer. Once again, we remain at your disposal for any other suggestions.

Reviewer #1: Abstract, Results, lines 39-41: please consider rephrasing this as it isn’t quite clear what differences were tested for.

It was modified as suggested. line 38: "Additionally, rhinovirus (hRV) was more frequent in pneumococcal carriers versus non-carriers (39% versus 21.7%, p = 0.012) and the presence of hBOV alone was associated with the absence of pneumococcal carriage (2.7% versus 10.8% p = 0.016). "

Abstract line 46: it is still not clear to me how these findings enhance our understanding of the relationship between these potentially pathogenic agents. There have been many other reports on the association between pneumococcus and respiratory pathogens so the authors should add additional detail to highlight was is novel about this.
It was modified as suggested. line 47:" This study represents one of the first investigations in Latin America to explore the relationship between respiratory viruses and pneumococcal carriage in a healthy child population."

Introduction – PNC and IPD are introduced twice.

It was modified as suggested.

Please review the text – the introduction should also be reviewed to improve the flow of the messaging.

After a careful analysis, we consider that the flow of messaging is in line with the work. We remain at your disposal for specific improvements.

Methods/Results – were siblings controlled for in the analyses?

Included in the results. line 148 (results): "The siblings were not clustered in the analyses due to the small number of participants in this condition (only ten participants)."

Results – please include a discussion of the demographic details of the 229 children enrolled that is presented in the table – at least highlight if there are any major differences between demographic groups. Please reference to the table in this section too.

The highlights are in the description in table 1 of the results and we have put in bold the p-values that were statistically significant

Results/Conclusion – with such a high frequency of samples generating high Ct values for virus (30-35), this is most likely historic infection as the authors recognize and may not represent active infection –

As described in the methodology, children with a diagnosis of acute respiratory disease or who had been hospitalized in the week before the sample was taken were excluded. And we believe it wasn't clear the cut off and including methodology line 126: “The analysis of symptoms at the time of collection with each virus investigated was carried out using two cut-offs: threshold (Ct) <30 (high levels only), and <35 (any level).”

it is perhaps evidence of prior infection which could be skewing associations or why there isn’t higher density of pneumococcus at those sampling moments. Did you look into any association between virus and pneumococcus Ct value?

As described in the methodology that pneumococcus was analyzed by bacterial culture (Ct results are not available)

We appreciate all the suggestions of reviewer 2 that, we are convinced of this, will improve our article We examined each of the suggestions in detail and did our best to achieve a satisfactory answer. Once again, we remain at your disposal for any other suggestions.

Reviewer #2: In general, the authors were very responsive to the comments and the manuscript reads much better. However, there are still a few issues to address with some of the new analyses.

Major comments:
Abstract – line 31 (of tracked version): the design should NOT be listed as a cross-sectional cohort study. This was a cohort study an cross-sectional analysis performed at baseline and a longitudinal analysis conducted with the FU data.

It was modified as suggested.

Methods – line 85: same comment – change to cohort study.

It was modified as suggested.
Methods – line 91: the authors now indicate that siblings were included but not what proportion of participants involved sibling pairs and therefore how many households are represented. If a large number of siblings were in fact enrolled, then there is clustering in the study population and this should be accounted for in the prevalence estimates and analyses.

line 149 (results): "The siblings were not clustered in the analyses due to the small number of participants that were in this condition (only 10 participants)."

Methods – lines 133-135: The multinomial regression needs to better described – what were the categories, what was the reference group, how were covariates selected for inclusion, etc.

It was modified as suggested. line 140: " Multinomial Logistic Regression reference group is No agent identified with three outcomes Pneumococcal carriage only, Respiratory virus detection only, Co-occurrence and was performed for the categories and Age in Months, Male Sex, Doesn't sleep alone, Spring, Summer, Fall, Winter, Any illness symptoms at the interview. "

Methods – line 136: the authors indicate that they used Bonferroni tests to adjust for multiple comparison but then indicate that a p<0.05 was considered statistically significant. What, if any, multiple comparisons were adjusted for and what Bonferroni-corrected p-value was considered statistically significant?

the Bonferroni test is the post-hoc test of the Kruskal-Wallis test, applied when comparing the age of the child between the microorganisms (Table 1): as p<0.001 in the Kruskal-Wallis test, two-by-two multiple comparisons were carried out to find out between which microorganisms there was a difference in age. If we had used ANOVA, we would have had a wider range of multiple comparison tests to choose from in addition to Bonferroni, such as Tukey, for example, but for the Kruskal-Wallis test, the option that corrects the type I error is Bonferroni.

"Results with a p-value <0.05 were considered statistically significant"

Refers to all the tests used, including the multiple comparisons via Bonferroni, since the software provides the p-value results already corrected. The p-values of the multiple comparisons are not shown in the table, only their result: Co-occurrence more novel than Only Pneumo and None.

Results – line 157: the authors indicated in their response that they provided a confidence interval for the prevalence but it does not appear to be included.

It was modified as suggested. line 159: "The prevalence of pneumococcal carriage was 63.7% (146/229, CI95%: 57.4% - 69.8%) for and respiratory viruses 49.34% (113/229, CI95%: 42.9% - 55.8%). Among those with pneumococcal carriage, 54.8% tested positive for respiratory viruses (80/146; p = 0.033, CI95%: 46.7% - 62.7%), resulting in a co-occurrence rate of 34.9% (80/229, CI95%: 29.0% - 41.3%) for both *S. pneumoniae* and any virus."

Discussion – lines 249-259: the new paragraph about hBOV is somewhat confusing:
- Line 249 – does the presence of SPN as the only pathogen present refer to findings in MEF?

It was modified as suggested. line 246: " Similarly, a Japanese study observed a positive correlation between the co-detection of pneumococcus and hBOV, where 85.7% (12/14) of children tested positive exclusively for both pathogens in otitis media fluid (MEF). However, in the nasopharynx, this correlation was not observed, as the proportion of pneumococci was similar between groups of individuals positive and negative for hBOV and with other pathogenic bacteria. Notably, pneumococcus was the only pathogen present in all but one of the co-detections in hBOV-positive cases, whereas in the nasopharynx, it was mixed with other pathogenic bacteria [53].

- Line 251 – add period after ‘identified’

It was modified as suggested.
- Line 251 – the sentence reads as if the children are acting as antagonists – please revise

It was modified as suggested. line 253:" It is essential to highlight that in children without serious diseases these microorganisms may have an antagonistic role for colonization in the nasopharynx, potentially suggesting protection of pneumococcus in the presence of hBOV alone."

It is essential to highlight that in children without serious diseases these microorganisms may have an antagonistic role for colonization in the nasopharynx


- Line 252 – the sentence suggests protection of pneumococcus in the absence of hBOV. Don't your results suggest the opposite, that there is protection from SPN when hBOV is present? but only when it is present alone?

It was modified as suggested. Line 256
- Line 253 – what migration is referred to here? Migration to MEF?

It was modified as suggested. Line 251 "This suggests that these pathogens migrate to their target site (MEF), with the nasopharynx not being the site where the etiologic agent is identified.

- Line 259 – provide references for the well-known relationship with co-infection and increased risk of severe SPN disease

It was modified as suggested. We include new references 54 and 55.

Table 1: the authors have a category called ‘self-report ethnicity’. I believe this should be ‘self-reported race’ instead. They also have an option for ‘Yellow/Indian’, which is not a term generally used – consider ‘Indigenous’ instead.

It was modified as suggested.

Table 2: For the p-value, it is unclear what the comparison is. Is it <30/35 vs. >30/35? if so, then the % with each symptom in the reference group needs to be shown in the table in order to interpret the results. For example, sore throat is associated with RNV<35 with 5.3% having sore throat, but is that a higher or lower % than observed in those with RNV>35?

We believe it wasn't clear the cut off and including methodology line 126: “The analysis of symptoms at the time of collection with each virus investigated was carried out using two cut-offs: threshold (Ct) <30 (high levels only), and <35 (any level).The ct >35 were considered negative.”

And the table 2 footnote: "ct >35 was negative.

Minor comments:
Abstract – line 37 & Results – line 157: change to 49.3% (one decimal place).

It was modified as suggested.
Methods – line 131-132: change ‘qualitative’ to ‘categorical’ and ‘quantitative’ to ‘continuous’

It was modified as suggested. Line 135.
Results – line 157: add ‘of’ and ‘was’ (the prevalence OF pneumococcal carriage WAS…)

It was modified as suggested.
Results – line 159: italicize S. pneumoniae

It was modified as suggested.
Discussion – line 210: 47.2% in the table

It was modified as suggested.
Discussion – line 212: change ‘findings’ to ‘symptoms’

It was modified as suggested.
Discussion – line 224: INF and PIV are introduced as new abbreviations – spell out first

the abbreviations were spelled out first in lines 124 and 125

Discussion – line 268: suggest tempering language and using ‘may have prevented development of diseases’

It was modified as suggested. Line 274

Table 4 – suggest combining with Table 5

It was modified as suggested.

| **Table 4. Incidence of medical appointments, clinical symptoms and diagnosis in 14 days of medical record analysis according to identified microorganism, Veranópolis/RS, Brazil, between 2018 and 2019.** | | | | | | |
| --- | --- | --- | --- | --- | --- | --- |
|  | **Total** | **Only Pneumococcal carriage** | **Only Respiratory viruses detection** | **Co-occurrence**¹ | **No agents identified** |  |
| n, (%) | 224 | 65 (29%) | 31 (13.8%) | 80 (35.7%) | 48 (21.4%) |  |
| Medical appointments | 43 | 11 (16.9%) | 8 (25.8%) | 14 (17.5%) | 10 (20.8%) |  |
| Any documented clinical symptoms within 14 days, n (%) | 41 (18.3%) ³ | 11 (16.9%) | 7 (22.6%) | 14 (17.5%) | 9 (18.8%) |  |
| Cough | 24 (10.71%) | 7 (10.8%) | 5 (16.1%) | 6 (7.5%) | 6 (12.5%) |  |
| Nasal congestion/ coryza | 18 (8.04%) | 7 (10.8%) | 2 (6.5%) | 4 (5%) | 5 (10.4%) |  |
| Fever | 13 (5.8%) | 5 (7.7%) | 2 (6.5%) | 2 (2.5%) | 4 (8.3%) |  |
| Sore throat | 8 (3.6%) | 4 (6.2%) | 0 (0) | 4 (5%) | 0 |  |
| Any diagnosis within 14 days, n (%) | 43 (19.2%) | 11 (16.9%) | 8 (25.8%) | 14 (17.5%) | 10 (20.8%) |  |
| Pneumonia | - | - | - | - | - |  |
| Tonsillopharyngitis | 8 (3.6%) | 6 (9.2%) | 1 (3.2%) | 1 (1.3%) | - |  |
| Sinusitis | 6 (2.7%) | 1 (1.5%) | 2 (3.2%) | 2 (2.5%) | 2 (4.2%) |  |
| AOM² | 2 (0.9%) | 2 (1.5%) | 0 | 1 (1.3%) | - |  |
| ARI | 23 (10.3%) | 4 (6.2%) | 4 (12.9%) | 8 (10%) | 7 (14.6%) |  |
| Asthma | - | - | - | - | - |  |
| Gastroenteritis | 8 (3.6%) | 1 (1.5%) | 2 (6.5%) | 3 (3.8%) | 2 (4.2%) |  |
| Not statistically significant by the chi-square or Fisher’s exact test.  ¹Co-occurrence = Pneumococcal carriage and respiratory virus detection  ²AOM = [acute otitis media](http://paperpile.com/b/1bNmpM/kEwi)  ³ No symptoms were reported at two medical appointments | | | | | | |

Table 5 – it is interesting that there isn't complete overlap in the “any documented clinical symptoms’ category and having a medical appt in Table 4. What accounts for the discrepancy?

We included the footnote “³ No symptoms were reported at two medical appointments”

S2 Table 1 – the current column (microorganisms) reporting n (%) for each covariate is not interpretable without the corresponding column for ‘no pathogen detected’.

It was modified as suggested.

S2 Table 2:
- Be consistent in the number of decimals presented in this table

It was modified as suggested.

- Remove the % in the top row labeled ‘medical records’ – these are row percents while the of the table presents column percents and it is confusing.

It was modified as suggested.

- There is a footnote outlining tests conducted and ‘* p<0.05 significant’, but it’s not clear if any comparisons are being made in this table and nothing is significant. If there are comparisons being made, please outline what they are and what the reference group is.

We removed it because there were no statistically significant variables.

S2 Table 3: While the authors have indicated the statistical test being conducted, it’s still not clear what comparison is being made. For example, there is an asterix on RSV for Fall – is this comparing the distribution of seasons between those that are RSV+ and RSV-? If so, this is only interpretable if we have the distribution for the RSV- group.

We compared, for each virus (lines), the frequency in the different seasons (columns). The Table 3 is now modified, and it is easier to find the association with RSV and fall.

**S2 Table 3. Detection of different respiratory viruses according to Seasons, Veranópolis/RS, Brazil, between 2018 and 2019.**

|  | **Summer** | **Fall** | **Winter** | **Spring** | ***p-value*** |
| --- | --- | --- | --- | --- | --- |
| Respiratory viruses,  n (%) | 6 ( 2.6%) | 102 (44.5%) | 97 (42.4%) | 24 (10.5%) | 17 (7.4%) |
| hRV | 2 (33.3%) | 38 (37.3%) | 30 (30.9%) | 5 (20.8%) | 0.451 |
| ADV | 0 (0%) | 13 (12.7%) | 8 (8.2%) | 2 (8.3%) | 0.590 |
| hBOV | 0 (0%) | 10 (9.8%) | 17 (17.5%) | 0 (0%) | 0.058 |
| RSV | 0 (0%) | **13 (12.7%)** | 4 (4.1%) | 0 (0%) | **0.043** |
| MPV | 0 (0%) | 11 (10.8%) | 4 (4.1%) | 2 (8.3%) | 0.293 |
| The chi-square or Fisher’s exact test | | |  |  |  |
| **p < 0.05 significant** | |  |  |  |  |
