# Supplementary material for: Nasopharyngeal carriage of Streptococcus pneumoniae among Brazilian children: Interplay with viral co-infection
Source: PLoS One. 2025 Jan 2;20(1):e0316444. doi: 10.1371/journal.pone.0316444 (PMC11694996; doi:10.1371/journal.pone.0316444)
Supplement: Attachment — Submitted filename: Response to Reviewers III.docx [file pone.0316444.s008.docx]

The authors of this manuscript would like to thank the editors and reviewers of the journal Plos One for the excellent progress. We believe that we have made a remarkable improvement. The indication of Editage was also very important in this progress towards publication. Thanks to everyone involved. Here are the changes to the manuscript detailed in this document:

Specific comments:

1. Line 39: Change hBOV to human bocavirus (hBOV)

It was modified as suggested.

2.	Line 126: Change “described” to “, as described”

It was modified as suggested.

3.	Line 141 – 144: This is an awkward run on sentence which need rephrasing for clarity.

It was modified as suggested.line 129: "The association between pneumococcal carriage and/or respiratory virus detection and age was assessed using the Kruskal–Wallis test followed by post-hoc Dunn’s pairwise tests and Bonferroni correction."

4.	Line 151: If 6 were excluded, this should be 223. In figure 1, 5 were excluded, which would be 224. Please make sure that the numbers are consistent throughout the manuscript.

we were wrong to write 6 because there were 5 children excluded from the medical records

5.	Line 160 – 161: Change “… for and respiratory viruses 49.3% (113/229, CI95%: 42.9% - 55.8%).” To “…and 49.3% (113/229, CI95%: 42.9% - 55.8%) for respiratory viruses.”

It was modified as suggested.

6.	Line 163: Delete “for both S. pneumoniae and any virus”

It was modified as suggested.

7.	Table 1: Please include Female number for ease of reading.

It was modified as suggested: "Table 1"

| **Female Sex** | 114 (49.8%) | 36 (54.5%) | 16 (48.5%) | 29 (36.2%) | 33 (66%) | **-** |  |
| --- | --- | --- | --- | --- | --- | --- | --- |

8.	Line 172: Change “associated with co-occurrence” to “associated with higher rate of co-occurrence”

It was modified as suggested.

9.	Line 181 – 183: This is an awkward sentence, please rephrase for clarity.

It was modified as suggested. line 173: "Various viruses were detected in children with pneumococcal infections. A relationship was observed between total hRV (alone + co-detection) 39% (57/146, p = 0.011) and the detection of any respiratory virus 54.8% (80/146, p = 0.040) with pneumococcal carriage (Table 3). Additionally, the presence of hBOV alone was associated with the absence of pneumococcal carriage (10.8%; p = 0.016). All samples tested negative for INF A and B, as well as for hPIV 1, 2, and 3."

10. Table 3: Do the authors of the information of co-detection of viruses? It maybe helpful to have more details instead of just total number, for example hRV + ADV or hRV + RSV?

We agree with the request and have included a description of the “total” (viral co-occurrence), but the table was too long. That's why we've built an additional **Table S2** in Supplementary Information 2:

| **Table S2. Frequency of respiratory viruses in children with and without pneumococcal carriage, Veranópolis/RS, Brazil, between 2018 and 2019.** | | | | | | | |
| --- | --- | --- | --- | --- | --- | --- | --- |
|  | **Respiratory virus detection** | **Pneumococcal carriage** | | | ***p-value*** | |  |
|  |  | **Yes (n=146)** | **No (n=83)** | |  |  |  |
| **hRV** | Alone (n = 51) | 38 (26%) | 13 (15.7%) | | 0.100 | |  |
|  | Total¹ (n = 75) | 57 (39%) | 18 (21.7%) | | **0.011** | |  |
|  | Virus Co-occurrence ( n = 24)  hRV + BOV (n = 1)  hRV + ADV (n = 4)  hRV + ADV + BOV (n = 2)  hRV + ADV + MPV (n = 2)  hRV + ADV + RSV (n = 1)  hRV + MPV (n = 5)  hRV + MPV + BOV (n = 4)  hRV + RSV (n = 2)  hRV + RSV + MPV (n = 1)  hRV + RSV + BOV (n = 1)  hRV + ADV + MPV + RSV (n = 1) |  |  | |  | |  |
| **ADV** | Alone (n = 9) | 6 (4.1%) | 3 (3.6%) | | 1.000 | |  |
|  | Total¹ (n = 23) | 18 (12.3%) | 5 (6%) | | 0.195 | |  |
|  | Virus Co-occurrence ( n = 14)  hRV + ADV (n = 4)  hRV + ADV + BOV (n = 2)  hRV + ADV + MPV (n = 2)  hRV + ADV + RSV (n = 1)  hRV + ADV + MPV + RSV (n = 1)  ADV + BOV (n = 2)  ADV + BOV + RSV (n = 1)  ADV + BOV + RSV + MPV (n = 1) |  |  | |  | |  |
| **hBOV** | Alone (n = 13) | 4 (2.7%) | 9 (10.8%) | | **0.016** | |  |
|  | Total¹ (n = 27) | 16 (11%) | 11 (13.3%) | | 0.761 | |  |
|  | Virus Co-occurrence ( n = 14)  hRV + BOV (n = 1)  hRV + ADV + BOV (n = 2)  hRV + MPV + BOV (n = 4)  hRV + RSV + BOV (n = 1)  ADV + BOV (n = 2)  ADV + BOV + RSV (n = 1)  ADV + BOV + RSV + MPV (n = 1)  BOV + MPV (n = 1)  BOV + RSV (n = 1) |  |  | |  | |  |
| **RSV** | Alone (n = 8) | 7 (4.8%) | 1(1.2%) | | 0.264 | |  |
|  | Total¹ (n = 17) | 14 (9.6%) | 3 (3.6%) | | 0.163 | |  |
|  | Virus Co-occurrence ( n = 9)  hRV + RSV (n = 2)  hRV + RSV + MPV (n = 1)  hRV + RSV + BOV (n = 1)  hRV + ADV + RSV (n = 1)  hRV + ADV + MPV + RSV (n = 1)  ADV + BOV + RSV (n = 1)  ADV + BOV + RSV + MPV (n = 1)  BOV + RSV (n = 1) |  |  | |  | |  |
| **MPV** | Alone (n = 2) | 1 (0.7%) | 1 (1.2%) | | 1.000 | |  |
|  | Total¹ (n = 17)  Virus Co-occurrence ( n = 15) | 13 (8.9%) | 4 (4.8%) | | 0.384 | |  |
|  | hRV + ADV + MPV (n = 2)  hRV + MPV (n = 5)  hRV + MPV + BOV (n = 4)  hRV + RSV + MPV (n = 1)  hRV + ADV + MPV + RSV (n = 1)  ADV + BOV + RSV + MPV (n = 1)  BOV + MPV (n = 1) |  |  | |  | |  |
| **Any respiratory virus (n=113)** | | 80 (54.8%) | | 33 (39.7%) | | **0.040** | |
| The chi-square or Fisher’s exact test  **p < 0.05 significant**  ¹ Total = alone + co-detection with another respiratory virus | | | |  | |  | |

11. Line 204: Change “demonstrate” to “revealed”

It was modified as suggested.

12. Line 206 – 207: This is an awkward sentence, not sure what the authors wish to convey.

It was modified as suggested. line 184: "However, no statistically significant correlation was found between pneumococcal carriage, respiratory virus detection, or co-occurrence in the medical appointments of the participants 14 days after specimen collection (Table 4)"

13. Line 220: Not sure the significance and/or relevance of this sentence. Please clarify.

It was modified as suggested. line 168: "The only symptom associated with the occurrence of any virus was sore throat with any hRV Ct value (p < 0.05)."

14. Line 256: This sentence missed a period at the end.

It was modified as suggested.

15. Line 256 – 257: This sentence needs rephrasing for clarity.

It was modified as suggested. line 246: "However, interpretation of the results of both studies was limited by the small number of patients included."

Reviewer #1: I commend the authors for the work they have put in to further update their manuscript. I only have a couple more comments:

I believe line 38 where it is talking about respiratory viruses in carriers is also missing a ‘versus non-carriers’. The ‘versus non-carriers’ could move from line 39 to 38 to introduce this comparison and carry that through the subsequent comparisons.
It was modified as suggested.

It is still not entirely clear how the results enhance our understanding (line 47). That sentence could since the authors have added a new sentence about the study after that.

We thank the reviewer's comment and deleted the sentence: "These findings enhance our understanding of the relationship between these potentially pathogenic agents."

Line 165 – it is not clear in the text to what the p-value represents.

It was modified as suggested. line 154: "As shown in Table 1, children who tested positive for viral infection, with or without the co-occurrence of pneumococcus, were younger than pneumococcal carriers (31 and 33 months vs. 42 months; p < 0.001)."

Reviewer #2: The authors have adequately addressed most comments. There are two outstanding comments:
Table 2: while the authors have clarified that they performed the analysis using two different cutoffs, the table only presents one of the proportions from the comparison being made. Presumably, the comparison being made and referenced by the p-value is the % symptomatic with virus <30 (or <35) compared to the % symptomatic with virus >=30 (or >=35). Given that only RNV is significant at ct<35, I suggest presenting the comparison of <35 vs. >=35 in Table 2 and creating a supplemental table to show the results for <30 vs. >=30 and just refer to the results being consistent at this lower ct cutoff in the text.

Following the suggestions of the reviewers, we explored the cutoff values and the associations with symptoms. We can present only results of the Ct 35, as suggested (this was the original version of the table) and create a new supplementary table. However, we consider that Table 2, with different cutoffs, is necessary at this point.

Also, combine the first two footnotes (e.g., * p<0.05 significant by chi-square or Fisher’s exact test) – the same should be done for Table 1 as well. It was modified as suggested.

Methods - line 138-141: Can the authors clarify if the age comparison using the Kruskal-Wallis was followed by post-hoc Dunn’s test with Bonferroni adjustment. The sentences should be clarified to indicate what was done (e.g., The association between pneumococcal carriage and age was conducted using a Kruskal-Wallis test and followed with post-hoc Dunn’s pairwise tests and Bonferroni correction. The association of pneumococcal carriage and demographic data other than age, symptoms,…).

It was modified as suggested. line:129 "The association between pneumococcal carriage and/or respiratory virus detection and age was assessed using the Kruskal–Wallis test followed by post-hoc Dunn’s pairwise tests and Bonferroni correction."

Additional minor comments:
Abstract – line 31 (tracked version): modify sentence to indicate 229 nasopharyngeal samples collected from children aged 18-59 months at baseline were included.

It was modified as suggested

Methods – line 88-101: modify sentence in line 88 to delete ‘and cross-sectional analysis’.

It was modified as suggested

Modify line 98 to indicate ‘at enrollment, health professionals trained for the study collected a nasopharyngeal specimen and conducted structure…..’.

It was modified as suggested

Methods – line 127-129: move new text to Statistical Analysis section and describe this analysis (chi-square or Fisher’s exact test).

It was modified as suggested.line 136: "A chi-square or Fisher's exact test was carried out on the symptoms at the time of collection, and each virus was investigated using two cut-off points: Ct <30 (high levels only) and <35 (any level); Ct >35 was considered negative."

Methods – line 141-144: revise the sentence regarding multinomial regression for English (e.g., The multinomial logistic regression included four outcome categories including ‘agent identified’ (reference group), ‘pneumococcal carriage only’, ‘ respiratory virus detection only’, and ‘co-occurrence’. Exposure variables included age in months, male sex, does not sleep alone, season, and any illness symptoms at enrollment.)

It was modified as suggested. line 33: "The multinomial logistic regression included four outcome categories including ‘agent identified’ (reference group), ‘pneumococcal carriage only, ‘ ‘respiratory virus detection only,’ and ‘co-occurrence.’ The exposure variables included age (months), male sex, lack of sleep, season, and any illness symptoms at enrollment. "

Methods – line 135: add abbreviation for confidence level (CI) as it is used in the results.

It was modified as suggested.
Results – line 135: clarify that it is ‘PNC prevalence at enrollment’

It was modified as suggested: "The sample size was calculated considering the Brazilian PNC prevalence of 62.3%"
Results – line 164: fix grammar leading to prevalence for respiratory viruses

It was modified as suggested.
Results – line 165: a p-value is provided for the % with respiratory viruses among those with PNC but it is not interpretable unless the % among those without PNC is provided – either remove or provide the % (e.g., p=0.033 compared to X% among those without PNC).

It was modified as suggested. "The prevalence of pneumococcal carriage was 63.7% (146/229, CI 95%: 57.4–69.8%) and 49.3% (113/229, CI 95%: 42.9–55.8%) for respiratory viruses. Respiratory viruses were more frequently found among pneumococcal carriers (54.4% (80/146) vs. non-carriers [39.7% (33/83); p = 0.033] ). CI95%: 46.7–62.7%)."

Results – line 166: italicize S. pneumoniae

It was modified as suggested.

Discussion – line 261 – put back in the period after ‘hBOV alone’.
It was modified as suggested.

References - italicize S. pneumoniae throughout

It was modified as suggested.
