# Supplementary material for: Nasopharyngeal carriage of Streptococcus pneumoniae among Brazilian children: Interplay with viral co-infection
Source: PLoS One. 2025 Jan 2;20(1):e0316444. doi: 10.1371/journal.pone.0316444 (PMC11694996; doi:10.1371/journal.pone.0316444)
Supplement: Attachment — Submitted filename: rebuttal letter IV.docx [file pone.0316444.s009.docx]

The authors of this manuscript would like to thank the editors and reviewers of the journal Plos One for the excellent progress. Thanks to everyone involved. Here are the changes to the manuscript detailed in this document:

6. Review Comments to the Author

Reviewer #2: I thank the authors for addressing the comments. The manuscript is greatly improved. I have just one remaining comment regarding Table 2 that should be addressed before the manuscript can be accepted. The authors present two cutoffs (which is fine) but do not present the comparison group (which I believe is >35 for both for both cutoffs). Without presenting the results for >35, it is not possible to interpret the % provided. For example, 5.3% of RNV with >35 have a sore throat, but what is that being compared to? is that higher or lower than among those with >35? A column for >35 should be added for each pathogen.

It was modified as suggested: "Table 2"

| **Table 2. Frequency of clinical symptoms in children with detection of respiratory viruses by real-time PCR using the cut-off levels Cycle threshold (CT), Ct- value of < 30 (only high levels) and < 35 (any level), Veranópolis/RS, Brazil, between 2018 and 2019.** | | | | | | | | | | | | | | | | | | |  |
| --- | --- | --- | --- | --- | --- | --- | --- | --- | --- | --- | --- | --- | --- | --- | --- | --- | --- | --- | --- |
|  | Total | RNV | | | ADV | | | BOV | | | RSV | | | MPV | | | Any respiratory virus | | |
| Cycle Threshold (Ct) |  | <30 | <35 | > 35 | <30 | <35 | > 35 | <30 | <35 | >35 | <30 | <35 | >35 | <30 | <35 | >35 | <30 | <35 | >35 |
| n, (%) | 229 | 33 (14.4) | 75 (32.8) | 154 (67.2) | 9 (3.9) | 23 (10) | 206 (90) | 6 (2.6) | 27 (11.8) | 202 (88.2) | 3 (1.3) | 17 (7.4) | 212 (92.6) | 2 (0.9) | 17 (7.4) | 212 (92.6) | 47 (20.5) | 113 (49.3) | 116 (50.7) |
| Cough | 79 (34.5) | 11 (33.3) | 26 (34.7) | 53 (34.4) | 2 (22.2) | 5 (21.7) | 74 (35.9) | 0  (0) | 8 (29.6) | 71 (35.1) | 0  (0) | 4 (23.5) | 75 (35.4) | 0  (0) | 5 (29.4) | 74 (34.9) | 13 (27.7) | 38  (33.6) | 41 (35.3) |
| Nasal congestion/coryza | 108 (47.2) | 17 (51.5) | 35 (46.7) | 73 (47.4) | 3 (33.3) | 10 (43.5) | 98 (47.6) | 1 (16.7) | 10 (37) | 98 (48.5) | 0  (0) | 5 (29.4) | 103 (48.6) | 1 (50) | 7 (41.2) | 101 (47.6) | 21 (44.7) | 48  (42.5) | 60 (51.7) |
| Sore throat | 5 (2.2) | 2  (6.1) | **4 (5.3)** | 1 (0.6) | 0  (0) | 0  (0) | 5 (2.4) | 0  (0) | 0  (0) | 5 (2.5) | 0  (0) | 0  (0) | 5 (2.4) | 0  (0) | 0  (0) | 5 (2.4) | 2 (4.3) | 4  (3.5) | 1 (0.9) |
| p<0.05 significant by Chi-square or Fisher’s exact test | | | | | | | | | | | | | | | | | | |  |
| ct >35 was negative | | | |  |  | | | | |  |  |  |  |  |  |  |  |  |  |
